# Effects of platelet concentrates on implant stability and marginal bone loss: a systematic review and meta-analysis

**DOI:** 10.1186/s12903-021-01929-x

**Published:** 2021-11-12

**Authors:** Changxing Qu, Feng Luo, Guang Hong, Qianbing Wan

**Affiliations:** 1 State Key Laboratory of Oral Diseases, National Clinical Research Center for Oral Diseases, West China School of Stomatology, Sichuan University, Chengdu, 610041 China; 2 Liaison Center for Innovative Dentistry, Graduate School of Dentistry, Tohoku University, Sendai, Japan; 3 Department of Prosthetic Dentistry, Faculty of Dental Medicine, Airlangga University, Surabaya, Indonesia

**Keywords:** Platelet concentrates, Implant stability, Marginal bone loss, Systematic review, Meta-analysis

## Abstract

**Background:**

Osseointegration is essential for the success and stability of implants. Platelet concentrates were reported to enhance osseointegration and improve implant stability. The purpose of this review is to systematically analyze the effects of platelet concentrates on implant stability and marginal bone loss.

**Methods:**

Two researchers independently performed searches in the following databases (last searched on 21 July 2021): MEDLINE (PubMed), Cochrane Library, EMBASE, and Web of Science. In addition, a manual search was carried out on references of relevant reviews and initially included studies. All randomized controlled trials (RCTs) and controlled clinical trials (CCTs) on the application of platelet concentrates in the implant surgery procedure were included. The risk of bias of RCTs and CCTs were assessed with a revised Cochrane risk of bias tool for randomized trials (RoB 2.0) and the risk of bias in non-randomized studies—of interventions (ROBINS-I) tool, respectively. Meta-analyses on implant stability and marginal bone loss were conducted. Researchers used mean difference or standardized mean difference as the effect size and calculated the 95% confidence interval. In addition, subgroup analysis was performed based on the following factors: type of platelet concentrates, method of application, and study design.

**Results:**

Fourteen studies with 284 participants and 588 implants were included in the final analysis. 11 studies reported implant stability and 5 studies reported marginal bone level or marginal bone loss. 3 studies had high risk of bias. The meta-analysis results showed that platelet concentrates can significantly increase implant stability at 1 week (6 studies, 302 implants, MD 4.26, 95% CI 2.03–6.49, *P* < 0.001) and 4 weeks (8 studies, 373 implants, MD 0.67, 95% CI 0.46–0.88, *P* < 0.001) after insertion, significantly reduced marginal bone loss at 3 months after insertion (4 studies, 95 implants, mesial: MD − 0.33, 95% CI − 0.46 to − 0.20, *P* < 0.001; distal: MD − 0.38, 95% CI − 0.54 to − 0.22, *P* < 0.001). However, the improvement of implant stability at 12 weeks after insertion was limited (*P* = 0.10). Subgroup analysis showed that PRP did not significantly improve implant stability at 1 week and 4 weeks after insertion (*P* = 0.38, *P* = 0.17). Platelet concentrates only placed in the implant sites did not significantly improve implant stability at 1 week after insertion (*P* = 0.20).

**Conclusions:**

Platelet concentrates can significantly improve implant stability and reduce marginal bone loss in the short term. Large-scale studies with long follow‐up periods are required to explore their long-term effects and compare effects of different types.

***Trial registration*:**

This study was registered on PROSPERO, with the Registration Number being CRD42021270214.

**Supplementary Information:**

The online version contains supplementary material available at 10.1186/s12903-021-01929-x.

## Background

Osseointegration is the key to the success and stability of implants [1, 2]. It is defined as "the direct structural and functional connection between bone and the implant under the light microscope" [3]. The criteria for the success and influencing factors of osseointegration have been pointed out in some studies [4, 5]. Several methods have been introduced to enhance osseointegration and improve implant stability, including platelet concentrates [6].

Platelet concentrates are concentrated suspensions that consist of growth factors and platelets derived from blood [7, 8]. Their primary role is to promote tissues regeneration and wound healing [9]. Platelet concentrates include 3 different types: platelet-rich plasma (PRP), platelet-rich fibrin (PRF), and concentrated growth factor (CGF) [10]. PRP is the plasma fraction of autologous blood with higher platelet concentration as the first-generation platelet concentrate [11]. PRF is a fibrin matrix containing platelets, leukocytes, and growth factors as the second-generation concentrate [12]. As the latest generation product, CGF contains a higher concentration of growth factors and presents a denser network structure [13]. Platelet concentrates are prepared by centrifugation technology [14]. The process is controlled by the number of centrifugations, centrifugation time, rotate speed, and other factors [14]. The preparation process of PRF and CGF is simpler than that of PRP [15]. There are two ways to apply platelet concentrations [16, 17]. One is to dip the implant in platelet concentrates before insertion [17], and the other is to inject or insert them into the implant sites directly [16]. PRF and CGF are usually applied in the form of membranes [11, 16].

The role of platelet concentrates in promoting tissues healing is mainly dependent on many growth factors released [7]. These growth factors include platelet-derived growth factor (PDGF), transforming growth factor-β1 and β2 (TGF-β1, TGF-β2), vascular endothelial growth factor (VEGF), among others [18]. Platelet concentrates releasing these growth factors to induce angiogenesis [19], promote the proliferation and differentiation of osteoblasts [20]. Some research results showed that platelet concentrates could significantly increase implant stability [1, 21–24]. Pirpir et al. and Öncü et al. both reported that implant stability quotient (ISQ) in the platelet concentrates group was significantly higher than that in the control group [1, 21]. In addition, platelet concentrates can increase bone density and reduce marginal bone loss after implant insertion [22, 25].

In the relevant systematic reviews currently published, Fujioka-Kobayashi et al. conducted a meta-analysis of the effect of PRF in sinus elevation [26]. They did not analyze the effects of other platelet concentrates and their results cannot be appliable to the general clinical situation of implant therapy [26]. González-Serrano et al. only analyzed one outcome (MBL) and Miron et al. did not conduct a meta-analysis [27]. In addition, some randomized controlled trials published in the past five years have not been included in these studies [1, 16, 28]. Therefore, the purpose of this study is to systematically review the randomized controlled trials and controlled clinical trials of platelet concentrates in oral implantology and conduct a meta-analysis of the effects of platelet concentrates on implant stability and marginal bone loss.

## Methods

This systematic review and meta-analysis followed the PRISMA statement [29, 30]. This study was registered on PROSPERO, with the Registration Number being CRD42021270214.

### Focused question

The focused question is: Can platelet concentrates improve implant stability and reduce marginal bone loss?

### PICOS criteria

The PICOS (Patients, Intervention, Comparison, Outcome, and Study design) criteria of this systematic review are as follows:

Patients (P): Partially or completely edentulous patients receiving implant placement. There are no restrictions on age and gender.

Intervention (I): Use platelet concentrates during implant placement, including platelet-rich plasma (PRP), platelet-rich fibrin (PRF), and concentrated growth factors (CGF).

Comparison (C): No use of platelet concentrates and other products that promote bone tissue repair.

Outcome (O): Primary outcomes: Implant stability, estimated by implant stability quotient (ISQ) and periotest value (PTV). Secondary outcomes: Marginal bone loss (MBL), that is the change in the marginal bone height on the mesial or distal side of the implant.

Study design (S): Randomized Controlled Trials (RCTs) and Clinical Controlled Trials (CCTs).

### Eligibility criteria

Inclusion and exclusion criteria were set according to the PICOS criteria.

Inclusion criteria: (1) patients who received the placement of dental implants for dentition defects; (2) application of platelet concentrates in the surgical procedure; (3) reporting implant stability and MBL; 4) RCTs or CCTs;

Exclusion criteria: (1) participants including diabetic patients (some studies showed that affected peri-implant bone formation and reduction of implant stability might appear in diabetic patients [31–33]); (2) studies with maxillary sinus augmentation, extraction socket bone healing, and bone graft; (3) only reporting other outcomes, such as percentage of new bone formation, implant survival rate, etc.; (4) retrospective studies, case series, animal experiments, in vitro experiments, reviews, systematic reviews, and conference abstracts;

### Information sources

Two researchers (CQ and FL) conducted searches independently in the following databases: MEDLINE (PubMed), Cochrane Library, EMBASE, and web of science. The date when these sources were last searched was 21 July 2021. The database coverage was up to 21 July 2021. In addition, a manual search was carried out on references of the initially included articles and relevant reviews.

### Search strategy

The details of the search strategy for all databases are shown in Additional file 1. The database coverage was up to 21 July 2021 and the databases were last searched on 21 July 2021. The brief process of search strategy development was as follows: Search strategy was made according to the PICOS criteria. We read the search strategy section of several Cochrane systematic reviews concerning dental implants, platelet-rich fibrin, and related topics [34–37]. After referring to some terms in these studies, a draft search strategy was developed. It was continuously refined during several searches until the final version was formed.

### Selection process

Two researchers (CQ and FL) independently performed the initial screening by reading the title and abstract. Then the studies were screened for inclusion by reading full texts. PICOS criteria were followed in the screening process. If there is any disagreement between two researchers, discuss until consensus was achieved.

### Data collection process

Two researchers (CQ and FL) independently extracted data using a self‐developed data extraction form. If there is any discrepancy between the two researchers’ collected data, discuss until the last collection was reached.

### Data items

Implant stability was considered as the primary outcome. We only collected the results measured by resonance frequency analysis (RFA) and Periotest. The time frame of measurement was no less than 1 week. MBL was considered as the secondary outcome. Any radiographic measurement (based on Intra Oral Periapical radiographs, Cone beam Computer Tomography, or other radiological data) was eligible for inclusion. No restrictions were placed on the type of software for measurement. The time frame of measurement was no less than one month. Some studies may only report marginal bone levels at some time points. These results were also collected because they can often be converted to MBL.

Some studies may report results at multiple time points. These results were all considered for collection. Nevertheless, the length of follow-up was chosen when deciding which outcomes were enough to combine for synthesis and representative to explain the findings of the study.

Other collected data were as follows: Author, Year, Country, Study design, Sample size, Sex, Age, Type of platelet concentrates, Method of application, and Result.

### Assessment of risk of bias

For RCTs, researchers used a revised Cochrane risk of bias tool for randomized trials (RoB 2.0) to assess the risk of bias [38]. The specific domains are as follows: bias from the process of randomization, bias due to deviations from expected interventions, bias from missing data, bias from measurement of the outcome, and bias from selection of reported results. For CCTs, researchers employed the risk of bias in non-randomized studies—of interventions (ROBINS-I) tool to assess the risk of bias [39]. The specific domains are as follows: bias from confounding, bias from the process of participant selection, bias due to classification of interventions, bias due to deviations from expected interventions, bias from missing data, bias from measurement of outcomes, bias from selection of the reported result. Two researchers (CQ and FL) independently judged the risk of bias in each domain and recorded the reasons for the judgment. If there is any disagreement between researchers, discuss until consensus was achieved.

### Effect measures

For implant stability and MBL, researchers used mean difference (MD) as the effect size and calculated the 95% confidence interval. When different methods for the same outcome measured results, standardized mean difference (SMD) was applied as the effect size to eliminate the differences in methods. The significance level for statistical significance was 0.05.

### Synthesis methods

Before synthesis, data preprocessing was required in the following two situations: (1) PTV in the results of implant stability was converted to the opposite number. Different from ISQ, the smaller the PTV value, the higher the implant stability. (2) Some studies only reported marginal bone levels at some time points. The researchers converted marginal bone level at the time point into the MBL for a period of follow-up based on the baseline marginal bone level. Statistical heterogeneity among studies was assessed using Cochran’s Q test and Higgins inconsistency index (I^2^) test. When *P* > 0.05 in Cochran’s Q test and I^2^ < 50%, a fixed model was used, and a random model was used when *P* < 0.05 or I^2^ > 50%. When there were results of individual studies that significantly deviated from the overall effect size, researchers will attempt to remove data from that synthesis to reduce heterogeneity. The results of data synthesis were presented through the forest plots. In addition, researchers performed subgroup analysis based on the following factors: type of platelet concentrates (PRF, CGF, and PRP), method of application (dipping the implant in the liquid before insertion and only placed in the implant sites), and study design (RCT and CCT). All analyses were implemented in the Review Manager (Ver. 5.3).

The narrative analysis was performed for some studies whose data could not be used for meta-analysis.

## Results

### Study selection

**Fig. 1:**
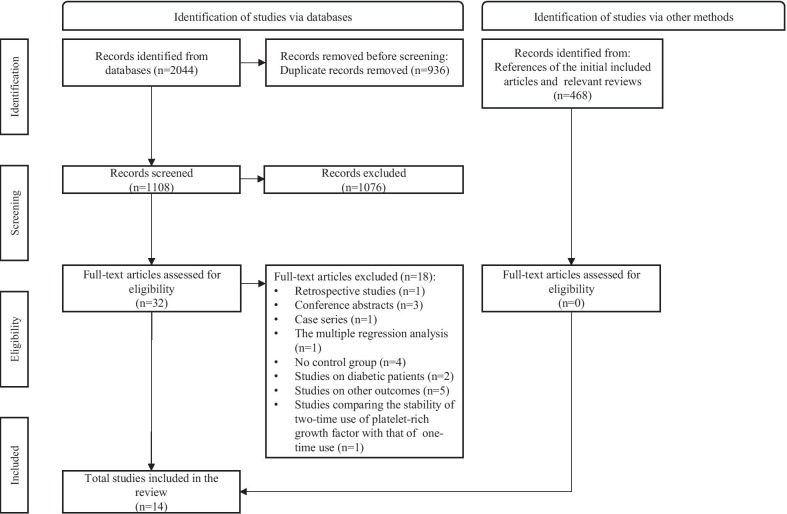
Flowchart of the study selection (PRISMA flow chart)

2044 articles were obtained through retrieval. After filtering out duplicates and articles with titles and abstracts that did not meet the criteria, 32 articles were included. By reading the complete text, 18 articles were screened out for some reasons. Finally, 14 articles were included [1, 6, 16, 17, 21–25, 28, 40–43], and 13 of them were meta-analyzed. The process of literature search and screening is shown in Fig. 1. Some studies that meet most inclusion criteria and reasons why they were excluded are listed in Additional file 2.

### Characteristics of the included studies

The included articles were published from 2005 to 2021, and 9 were published in the past 5 years [1, 6, 16, 22–24, 28, 42, 43]. 9 studies were RCTs [1, 17, 21, 25, 28, 42, 43] [22, 23] and 5 were CCTs [6, 16, 24, 40, 41]. For the platelet concentrates used in the studies, 8 studies used PRF [6, 21–25, 42, 43], 2 used CGF [1, 16], and 4 used PRP [17, 28, 40, 41]. For the method of application, researchers dipped implants in the platelet concentrate liquid before insertion in 7 studies [1, 17, 21, 24, 40, 41, 43]and only placed them in the implant sites in 7 studies [6, 16, 22, 23, 25, 28, 42]. For the primary outcome, ISQ was measured by resonance frequency analysis (RFA) in 8 studies [1, 6, 16, 21, 23, 24, 41, 42], and PTV was obtained by periotest in 2 studies [17, 22]. For the secondary outcome, 5 studies reported marginal bone level or marginal bone loss [17, 22, 25, 28, 43]. The characteristics of the included studies are shown in Table 1.

**Table 1:** Characteristics of included studies

References	Country	Study design	Sample size	Sex	Age	Type of platelet concentrates	Method of application	Outcome	Result
Alhussaini et al. [6]	Iraq	CCT^a^ (split mouth)	32	Female 59.4%	25–66, 48.6 ± 10.3	PRF^b^	PRF clot placed in the implant sites	Implant stability (ISQ^c^)	There was no statistically significant difference at 6 months (*P* = 0.348) and 12 months (*P* = 0.642)
Boora et al. [25]	India	RCT^d^	20	Female 25.0%	18–33, 24.6	PRF	PRF membrane placed in the implant sites	MBL^e^	The MBL of the test group was significantly lower than that of the control group within 3 months
Diana et al. [42]	India	RCT	31	Female 41.9%	28.5	PRF	PRF membrane placed in the implant sites	Implant stability (ISQ)	There was no statistically significant difference at 3 months (*P* = 0.645)
Ergun et al. [41]	Turkey	CCT	32	Female 53.1%	44.2 ± 12.5	PRP^f^	Dipping the implant in PRP liquid before insertion Injected into the implant socket	Implant stability (ISQ)	There was no statistically significant difference in all periods
Khan et al. [43]	India	RCT	17	Female 35.3%	NR	PRF	Dipping the implant in PRF liquid before insertion PRF membrane placed in the implant sites	MBL	There was no statistically significant Difference between the two groups at all follow-up intervals
Khan et al. [28]	Pakistan	RCT	12	NR^g^	NR	PRP	Injected into the implant socket	MBL	There was no statistically significant Difference between the two groups at all follow-up intervals
Kundu et al. [17]	India	RCT	30	Female 56.7%	18–56, 33.93 ± 11.25	PRP	Dipping the implant in PRP liquid before insertion	Implant stability (PTV^h^), MBL	For PTV, there was no statistically significant difference at 1 month (*P* = 0.107) and 3 months (*P* = 0.153) PRP had no significant effect on bone height changes at 1 month and 3 months (*P* > 0.05)
Monov et al. [40]	Austria	CCT (split mouth)	10	Female 60.0%	53–80, 67	PRP	Dipping the implant in PRP liquid before insertion Injected into the implant socket	Implant stability (Hz)	Except for the first week, there was no statistically significant difference between the two sides (*P* > 0.05)
Öncü et al. [21]	Turkey	RCT	20	Female 30.0%	44.2 ± 12.5	PRF	Dipping the implant in PRF liquid before insertion PRF membrane placed in the implant sites	Implant stability (ISQ)	ISQ in the test group was significantly higher than that in the control group at the end of the first week (*P* = 0.002) and the fourth week (*P* = 0.001)
Öncü et al. [22]	Turkey	RCT (split mouth)	26	Female 38.5%	40.2 ± 11.5	PRF	PRF membrane placed in the implant sites	Implant stability (PTV), MBL	Except for the third month, ISQ in the test side was significantly higher than that in the control side at 1 week and 1 month (*P* ≤ 0.002) PRF can significantly reduce marginal bone resorption after at least one year (*P* ≤ 0.05)
Koyuncu et al. [16]	Turkey	CCT	12	Female 58.3%	53–86	CGF^i^	CGF membrane placed in the implant sites	Implant stability (ISQ)	There was no statistically significant Difference between the two groups After 1, 2, and 4 weeks (*P* > 0.050)
Pirpir et al. [1]	Turkey	RCT	12	Female 58.3%	20–68, 44	CGF	Dipping the implant in CGF liquid before insertion CGF membrane placed in the implant sites	Implant stability (ISQ)	ISQ in the test group was significantly higher at week 1 and week 4 (*P* < 0.050)
Tabrizi et al. [23]	Iran	RCT (split mouth)	20	Female 55.0%	39.60 ± 6.74	PRF	PRF fibrin matrix placed in the implant sites	Implant stability (ISQ)	The PRF side got significantly higher ISQ at 2 weeks (*P* = 0.040), 4 weeks (*P* = 0.014), and 6 weeks (*P* = 0.027) after insertion
Torkzaban et al. [24]	Iran	CCT	10	Female 50.0%	26–60, 45.3	PRF	Dipping the implant in PRF liquid before insertion PRF membrane placed in the implant sites	Implant stability (ISQ)	ISQ in the test group was significantly higher at the end of the first week (*P* = 0.004) and the first month (*P* = 0.015)

^a^Controlled clinical trial

^b^Platelet-rich fibrin

^c^Implant stability quotient

^d^Randomized controlled trial

^e^Marginal bone loss

^f^Platelet-rich plasma

^g^Not reported

^h^Periotest value

^i^Concentrated growth factor

### Risk of bias

**Fig. 2:**
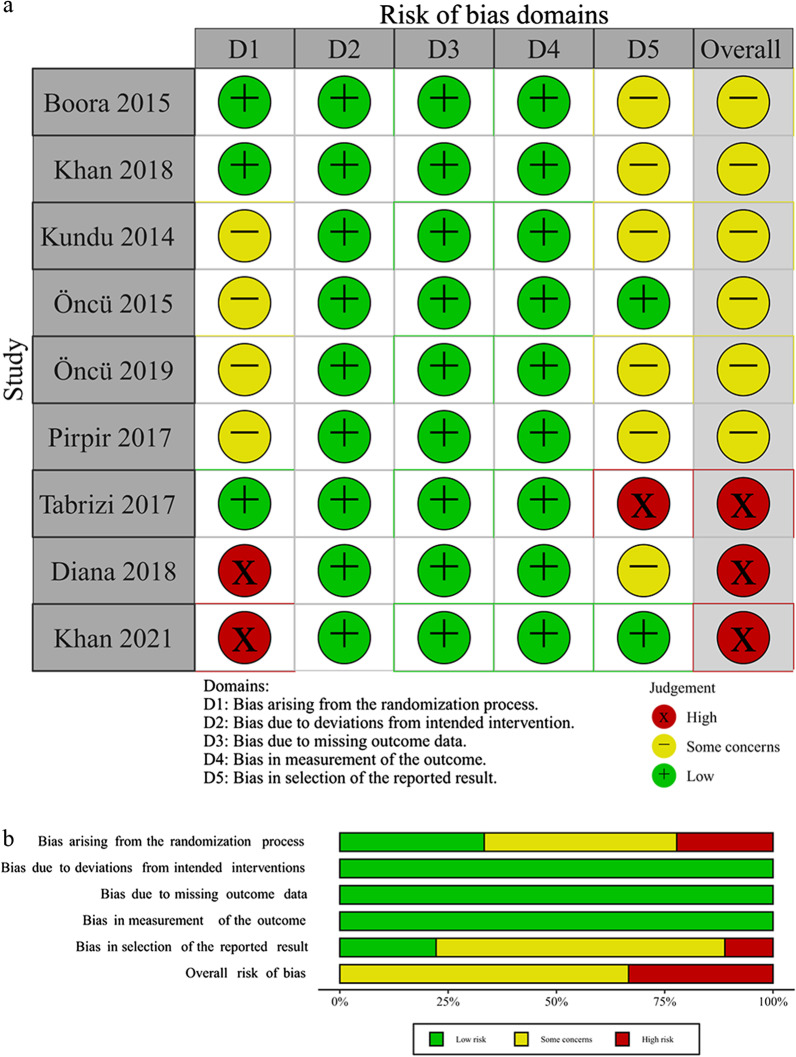
Risk of bias of RCTs: risk of bias summary (**a**) and risk of bias graph (**b**)

**Fig. 3:**
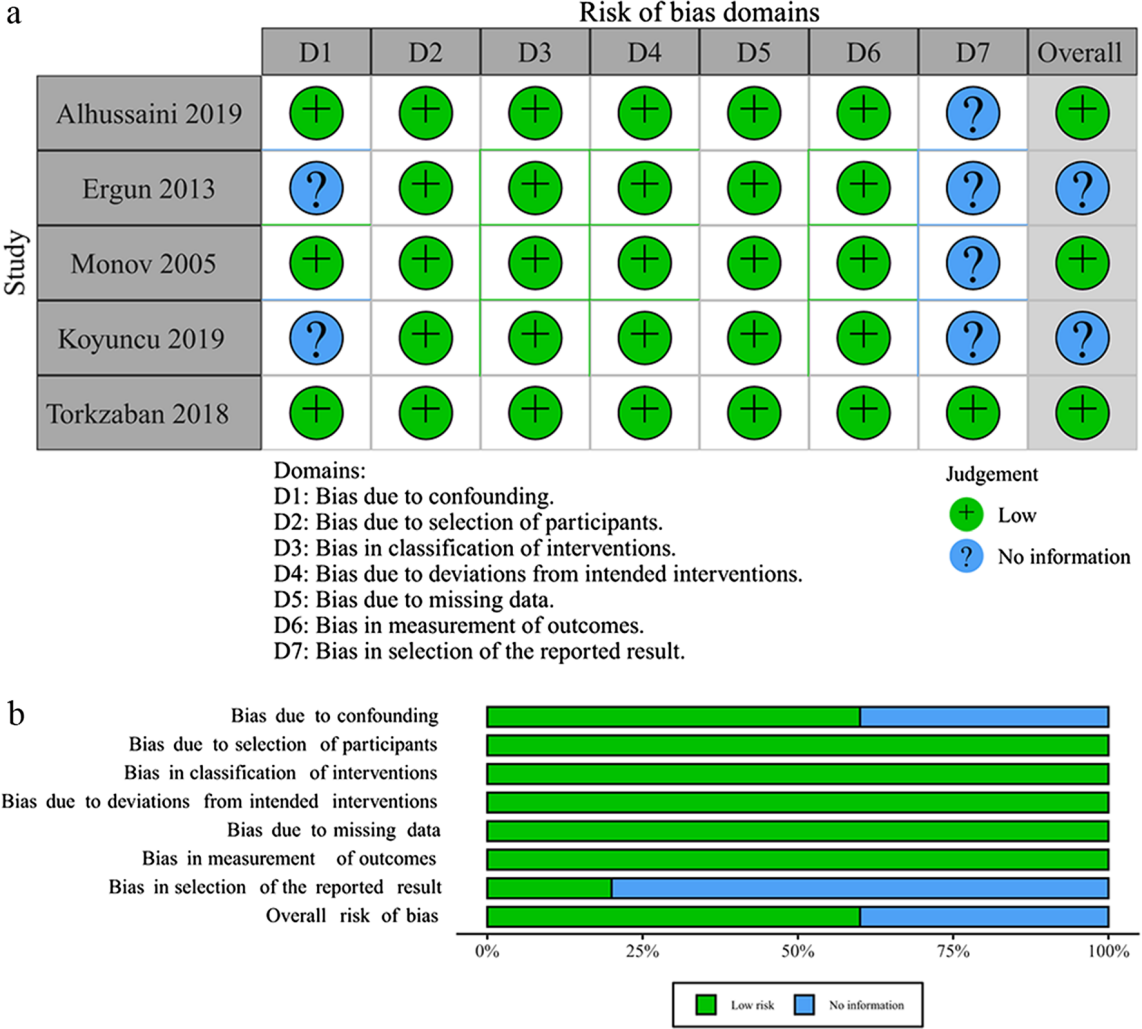
Risk of bias of CCTs: risk of bias summary (**a**) and risk of bias graph (**b**)

The results of the risk of bias assessment are shown in Figs. 2 and 3. The figures were made by Risk-of-bias VISualization (robvis) [44]. For RCTs, high risk of bias was seen in 3 studies. There was high risk of bias arising from the randomization process in 2 of the studies. Diana et al. grouped participants based on the order of arrival [42]. In the study of Khan et al., the random sequence was generated through non-probability sampling [28]. There was high risk of bias in the selection of the reported result in the study by Tabrizi et al. [23]. The results reported in this study did not completely match those in their protocol, lacking implant loss and bone height at 6 months after insertion. The risk of bias for the other 3 domains was all at low risk.

For CCTs, there was no information of bias due to confounding in the 2 studies. We considered the baseline bone quality as an important confounding factor. But Ergun et al. and Koyuncu et al. did not report it in the experimental group and the control group [16, 41]. In addition, we had access to only Torkzaban et al.’s study protocol [24]. So the risk of bias in the selection of the reported result in their study was low.

## The primary outcome—implant stability

Eleven studies reported on the effect of platelet concentrates on implant stability [1, 6, 16, 17, 21–24, 40–42]. Implant stability with a follow-up time of 1 week, 4 weeks, and 12 weeks were selected for meta-analysis.

### Implant stability at 1 week after insertion

Seven studies reported on implant stability 1 week after insertion [1, 16, 21, 22, 24, 40, 41]. Except for Monov et al.’s study (not report control group data) [40], a meta-analysis was performed on 6 studies reporting ISQ [1, 16, 21, 22, 24, 41]. A total of 302 implants was included in 6 studies. For platelet concentrates used, 3 studies used PRF [21, 22, 24], 2 studies used CGF [1, 16], and 1 study used PRP [41]. For the method of application, researchers dipped implants in platelet concentrate liquid before insertion in 4 studies [1, 21, 24, 41]. Researchers only placed them in the implant sites in 2 studies [16, 22]. 3 studies are RCTs [1, 21, 22]and 3 studies are CCTs [16, 24, 41]. For the risk of bias, 1 study had low risk of bias [16, 24, 41]. The overall risk of bias in the 3 studies was rated as “some concerns” [1, 21, 22]. There was no information on some risk of bias domains in the 2 studies [16, 41].

**Fig. 4:**
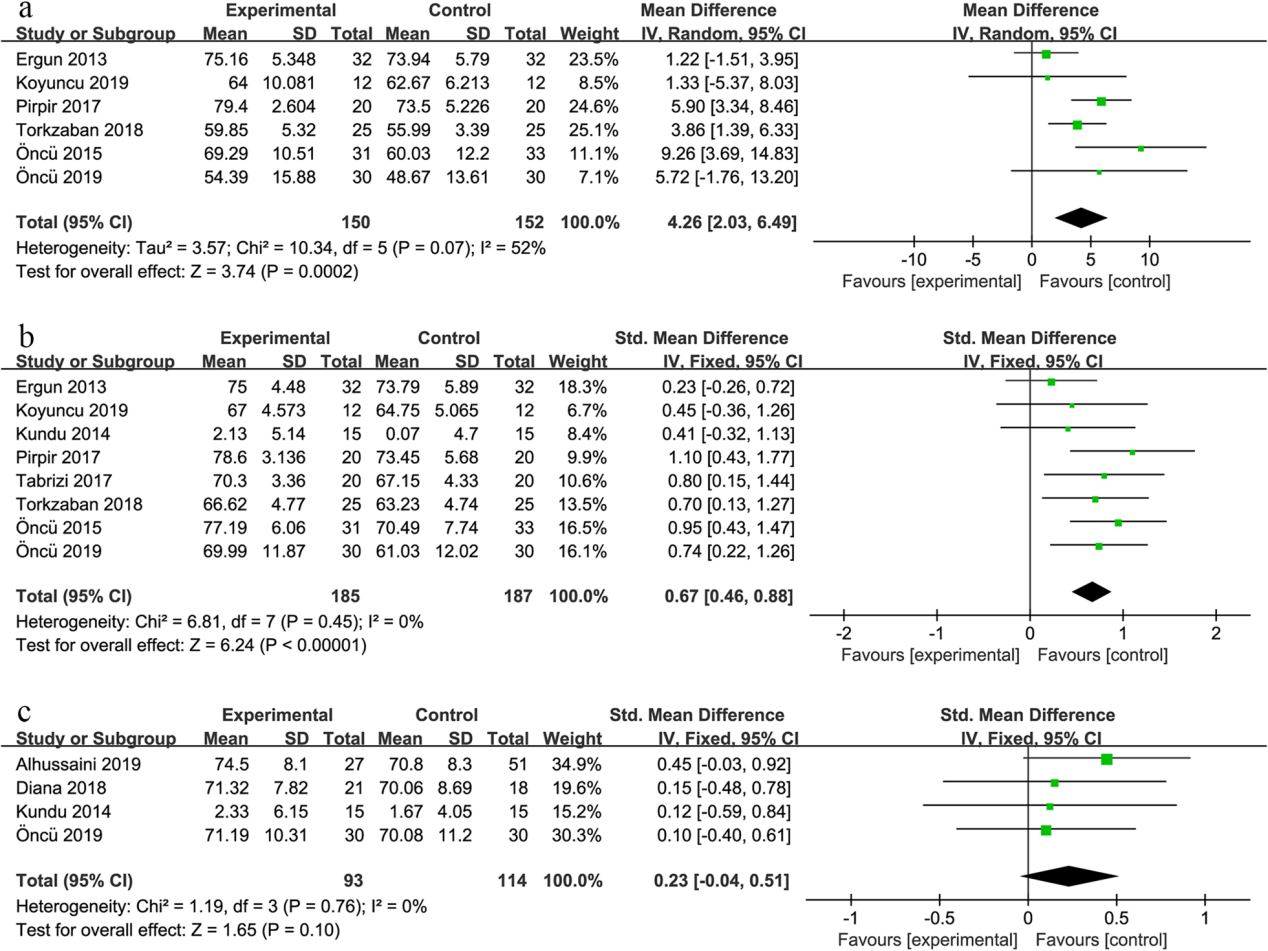
The forest plot of implant stability: at 1 week after insertion (**a**), at 4 weeks after insertion before (**b**), and at 12 weeks after insertion (**c**)

The forest plot (shown in Fig. 4a) showed that the heterogeneity of the six studies is a bit high (I^2^ = 52% and *P* = 0.07 in Cochran’s Q test). The MD between the experimental and control groups is 4.26, and the 95% confidence interval is (2.03, 6.49). The results are statistically significant (*P* < 0.001). This result indicates that platelet concentrates have a significant improvement in implant stability at 1 week after insertion.

The results of the subgroup analysis are shown in Table 2. For different types of platelet concentrates, implant stability was significantly higher in the PRF group and CGF group than the corresponding control groups (MD 5.53, 95% CI 2.19–8.88, *P* = 0.001; MD 4.71, 95% CI 0.77–8.64, *P* = 0.020). The application of PRP didn’t significantly improve implant stability (*P* = 0.38). For different application methods, the result of the group dipping implants in platelet concentrates was better than that of the control group (MD 4.51, 95% CI 1.79–7.22, *P* = 0.001). However, the result of another application method was not statistically significant (*P* = 0.20). Whatever the study design was, platelet concentrates can significantly improve stability (MD 6.42, 95% CI 4.20–8.64, *P* < 0.001; MD 2.56, 95% CI 0.71–4.41, *P* = 0.007). In addition, marked declines in heterogeneity were observed when we conducted subgroup analysis on the type of platelet concentrates and method of application.

**Table 2:** The subgroup analysis of meta-analysis on implant stability at 1 week after insertion

Group	Number of studies	SMD^a^ (MD^b^)	95% CI^c^	Heterogeneity	
				*P*_heterogeneity_	I^2^ (%)
*Type of platelet concentrates*					
PRF^d^	3	5.53	(2.19–8.88)	0.21	35
CGF^e^	2	4.71	(0.77–8.64)	0.21	36
PRP^f^	1	1.22	(− 1.51 to 3.95)	–	–
*Method of application*					
Dipping the implant in the liquid before insertion	4	4.51	(1.79–7.22)	0.02	68
Only placed in the implant sites	2	3.28	(− 1.71 to 8.27)	0.39	0
*Study design*					
RCT^g^	3	6.42	(4.20–8.64)	0.55	0
CCT^h^	3	2.56	(0.71–4.41)	0.35	5

^a^ standardized mean difference; ^b^ mean difference; ^c^ confidence Interval; ^d^ platelet-rich fibrin; ^e^ concentrated growth factor; ^f^ platelet-rich plasma; ^g^ randomized controlled trial; ^h^ clinical controlled trial

### Implant stability at 4 weeks after insertion

Nine studies reported on implant stability at 4 weeks after insertion [1, 16, 17, 21–24, 40, 41]. Except for Monov et al.’s study [40], a meta-analysis was performed on 8 studies. A total of 373 implants was included in these studies. 4 studies used PRF [16, 21–24, 41], 2 studies used CGF [1, 16], and 2 studies used PRP [17, 41]. Researchers dipped implants in platelet concentrate liquid in 5 studies [1, 17, 21, 24, 41]and chose another method in 3 studies [16, 22, 23]. 5 studies are RCTs [1, 17, 21–23] and 3 studies are CCTs [16, 24, 41]. There was no high risk of bias in these studies.

7 studies obtained ISQ measured by RFA [1, 16, 21–24, 41], and one study obtained PTV measured by periotest [17]. To eliminate differences in measurement methods, SMD was used as the overall effect size. Given the correlation between PTV and stability (the smaller the PTV, the greater the stability), the opposite of PTV was included in the meta-analysis. The Forest plot (shown in Fig. 4b) showed low heterogeneity (I^2^ = 0% and *P* = 0.45 in Cochran’s Q test) among the eight studies. Overall platelet concentrates can significantly improve implant stability at 4 weeks after insertion (*P* < 0.001; SMD 0.67; 95% CI 0.46–0.88).

The results of the subgroup analysis are shown in Table 3. Implant stability was significantly higher in the PRF group and CGF group than the corresponding control groups (SMD 0.80, 95% CI 0.52–1.08, *P* < 0.001; SMD 0.84, 95% CI 0.32–1.35, *P* = 0.001). The difference was not significant in the PRP group (*P* = 0.17). The results of other subgroups were statistically significant.

**Table 3:** The subgroup analysis of meta-analysis on implant stability at 4 weeks after insertion

Group	Number of studies	SMD^a^ (MD^b^)	95% CI^c^	Heterogeneity	
				*P*_heterogeneity_	I^2^ (%)
*Type of platelet concentrates*					
PRF^d^	4	0.80	(0.52–1.08)	0.92	0
CGF^e^	2	0.84	(0.32–1.35)	0.23	32
PRP^f^	2	0.28	(− 0.12 to 0.69)	0.69	0
*Method of application*					
Dipping the implant in the liquid before insertion	5	0.65	(0.40–0.91)	0.18	36
Only placed in the implant sites	3	0.70	(0.34–1.06)	0.79	0
*Study design*					
RCT^g^	5	0.82	(0.55–1.09)	0.69	0
CCT^h^	3	0.43	(0.09–0.77)	0.47	0

^a^Standardized mean difference

^b^Mean difference

^c^Confidence Interval

^d^Platelet-rich fibrin

^e^Concentrated growth factor

^f^Platelet-rich plasma

^g^Randomized controlled trial

^h^Clinical controlled trial

### Implant stability at 12 weeks after insertion

Four studies reported on the stability of the implants at 12 weeks after insertion [6, 17, 22, 42]. 207 implants were included in these studies. 3 studies used PRF [6, 22, 42] and 1 study used PRP [17]. Researchers dipped implants in platelet concentrate liquid before insertion in 1 study [17] and only placed concentrates in the implant sites in 3 studies [6, 22, 42]. One study had high risk of bias arising from the randomization process [42].

Three studies reported ISQ [6, 22, 42], and one reported PTV [17], so SMD was used as the overall effect size. The Forest plot (shown in Fig. 4c) showed a low heterogeneity of studies (I^2^ = 0% and *P* = 0.76 in Cochran’s Q test). The results showed limited improvement of platelet concentrates on implant stability at 12 weeks after insertion (*P* = 0.10; SMD 0.23; 95% CI − 0.04 to 0.51). Due to the limitation of the number of studies, subgroup analysis was not carried out on this outcome.

## The secondary outcome—MBL

Five studies reported the effect of platelet concentrates on marginal bone level or marginal bone loss [17, 22, 25, 28, 43]. A Meta-analysis was conducted on MBL with a follow-up of 3 months.

4 studies in the meta-analysis included 95 implants. 2 studies used PRF [25, 43] and 2 studies used PRP [17, 28]. Researchers dipped implants in platelet concentrate liquid before insertion in 2 studies [17, 43] and only placed concentrates in the implant sites in 2 studies [25, 28]. All studies were RCTs. The study by Khan et al. had high risk of bias because the random sequence was generated through non-probability sampling [28].

Researchers processed the data in the study by Kundu et al. to obtain MBL since they only reported marginal bone levels [17]. Öncü et al. only reported MBL 12 months after insertion, so it was not included in the meta-analysis [22]. Their results showed significantly lower MBL on the test side than on the control side (*P* ≤ 0.05).

**Fig. 5:**
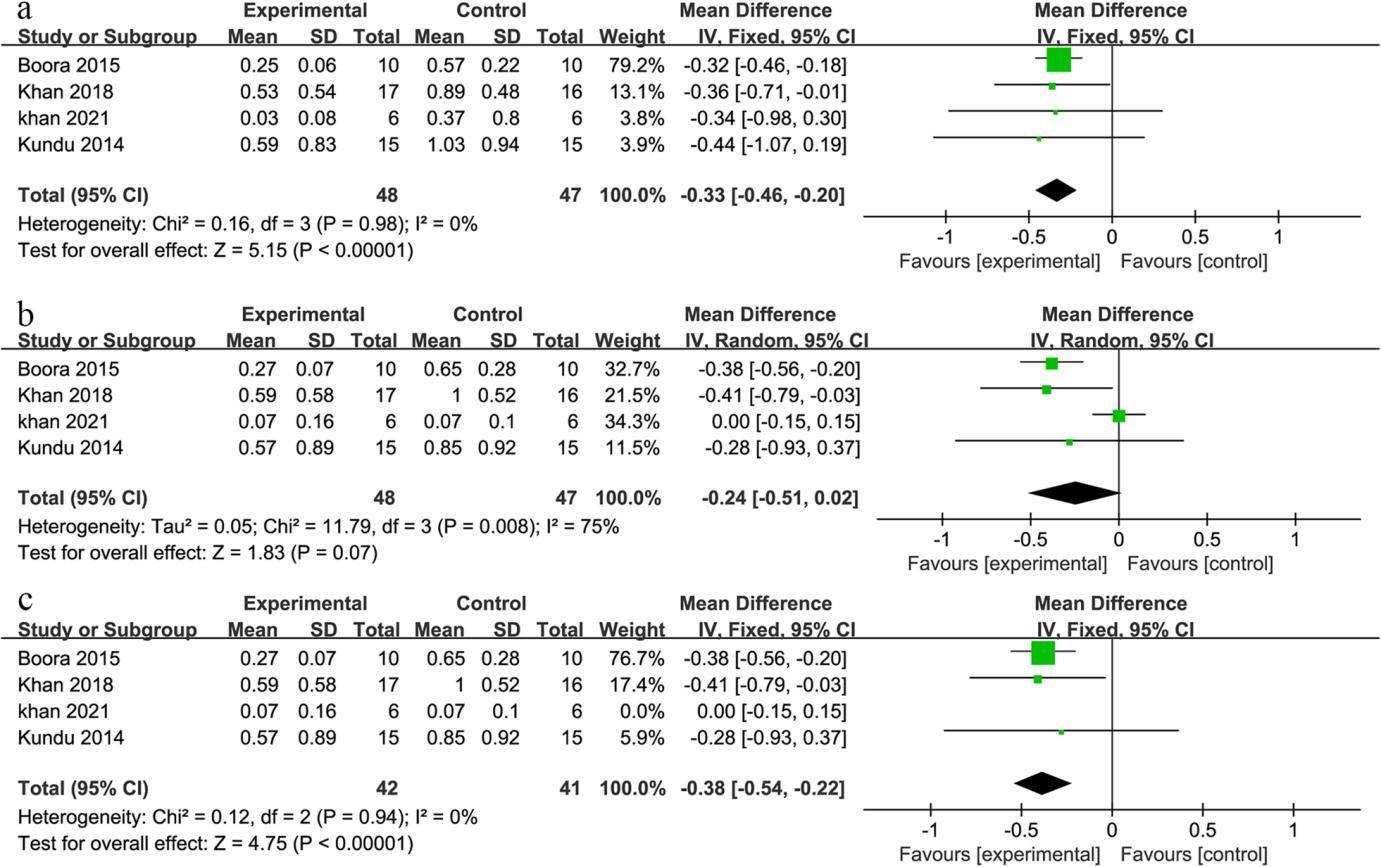
The forest plot of MBL at 3 months after insertion: on the mesial side of the implant (**a**), on the distal side of the implant before (**b**) and after (**c**) removing Khan et al.'s data

For MBL on the mesial side of the implant, the forest plot (shown in Fig. 5a) showed low heterogeneity of the study (I^2^ = 0% and *P* = 0.98 in Cochran’s Q test) and statistically significant results (MD − 0.33, 95% CI − 0.46 to − 0.20, *P* < 0.001).

For MBL on the distal side of the implant, the forest plot (shown in Fig. 5b) showed that the heterogeneity of the study is too high (I^2^ = 75% and *P* = 0.008 in Cochran’s Q test), even if a random model has been used. It was observed that the result of the study by Khan et al. deviated from the overall effect size [28], so researchers tried to remove it. The remaining 3 studies (shown in Fig. 5c) had low heterogeneity (I^2^ = 0% and *P* = 0.94 in Cochran’s Q test). The MD was − 0.38, and the 95% confidence interval was (− 0.54, − 0.22). The results were statistically significant (*P* < 0.001). Based on the above results, platelet concentrates can significantly reduce MBL at 3 months after insertion. Due to the limitation of the number of studies, subgroup analysis was not conducted on this outcome.

## Discussion

In this systematic review and meta-analysis, researchers analyzed the effect of the clinical application of platelet concentrates in terms of implant stability and MBL. The results showed that platelet concentrates significantly improved implant stability at 1 week and 4 weeks after insertion and reduced MBL at 3 months after insertion, although the improvement in implant stability at 12 weeks after insertion was limited. In addition, subgroup analysis showed that PRP had a limited effect on implant stability at 1 week and 4 weeks after insertion. The method of only placing platelet concentrates in the implant sites also had certain limitations. In general, platelet concentrates have a significant effect on improving implant stability and reducing MBL.

This result is consistent with the findings of some systematic reviews. Strauss et al. comprehensively analyzed the results of studies by Öncü et al., and Tabrizi et al. [21, 23, 45]. 2 studies all reported a significant increase in the ISQ value of the PRF group. Strauss et al. argued that PRF could promote the initial osseointegration process and increase implant stability. González-Serrano et al. conducted a meta-analysis evaluating MBL at 6 months and 12 months after insertion [27]. The analysis of the 2 outcomes included 3 studies respectively and the heterogeneity was both low (I^2^ = 0% and *P* = 0.72 in Cochran’s Q test; I^2^ = 0% and *P* = 0.65 in Cochran’s Q test). The results showed that MBL at 6 months and 12 months after insertion were significantly lower in the platelet concentrate group than the control group (MD − 0.50, 95% CI [− 0.57, − 0.43], *P* < 0.00001; MD − 0.50, 95%CI [− 0.57, − 0.43], *P* < 0.00001). The results of another systematic review are different from those of studies. Fujioka-Kobayashi et al. argued that PRF plays a lesser role in bone regeneration, sinus elevation, and implant therapy [26]. Nevertheless, all included studies (n = 2) that reported results of implant stability showed that PRF can slightly enhance primary implant stability.

The effect is mainly achieved by a large number of released growth factors. These growth factors include PDGF, TGF-β1 and β2, VEGF, fibroblast growth factor (FGF), bone morphogenetic protein (BMP), and insulin-like growth factor (IGF) [46–48]. Several in vitro studies have demonstrated the role of these growth factors in promoting bone regeneration and repair. He et al. cultured rat cranial osteoblasts using PRF and PRP and measured the amount of platelet-derived growth factor-AB (PDGF-AB) and transforming growth factor-1 (TGF-1) released by these platelet concentrates [49]. The results showed that PDGF-AB and TGF-1 could promote the synthesis of collagen, a skeleton for calcium deposition to promote bone formation. Ortolani et al. evaluated the effect of the combination of PDGF and insulin-like growth factor-1 (IGF-1) on implant osseointegration in rabbit femurs [50]. Their results showed that those growth factors promote the deposition of fibroblastic tissue and osteogenesis at the implant site. Chang et al. used an adenoviral vector encoding platelet-derived growth factor-B (PDGF-B) in dental implants from rats with alveolar bone defects and demonstrated that PDGF-B could significantly promote bone repair [51].

The results of subgroup analysis showed that PRF and CGF had significant effects while PRP did not. Their differences in releasing growth factors and promoting bone repair were evident in some studies. PRF releases growth factors gradually and can maintain their activity for a more extended period compared to PRP. In the study by He et al., PRF promoted the proliferation and differentiation of rat cranial osteoblasts more consistently and strongly than PRP [49]. CGF, the third-generation platelet concentrate, contains more growth factors than others and can release them for at least 13 days [52]. Some studies suggested that it may be more effective than PRP and PRF in promoting osteogenesis [53, 54]. Wei et al. found that Osterix mRNA expression levels were significantly higher in the CGF group than in the PRF group [54]. The promotion of osteoblast proliferation was more potent than that of PRF. As Lee et al. reported, the number of osteoblasts in CGF, both in the 10% and 50% preparations, was significantly greater than that in PRF [53].

Our study has proved the effect of platelet concentrates in the short term. Due to the lack of long follow-up studies, their long-term effect is still unknown. For implant stability, we reported a slight improvement in implant stability at 12 weeks after insertion. Moreover, the included studies all reported no significant difference in stability after 12 weeks between the experimental group and the control group. Ergun et al. reported that longer-term effects (6, 12, 24, and 36 months after insertion) were unsatisfactory [41]. For MBL, platelet concentrate still had a positive effect at 12 months after insertion in a systematic review and the study by Öncü et al. [22, 27]. However, a retrospective study reported no significant difference in MBL between the PRP group and the control group for a longer follow-up period (5 years) [48]. In addition, 37 patients with implants were followed up for an average of 13 years in the study by Attia et al. [55]. Among them, 17 patients received PRP and 20 patients did not take any treatment. The results showed that PRP could not significantly improve the long-term cumulative survival rate of implants.

The above studies suggested that the long-term effects of platelet concentrates may be limited. We believe that this is related to the duration of growth factors released from platelet concentrates. An in vitro study measured the number of growth factors released by advanced platelet-rich fibrin (A-PRF), injected platelet-rich fibrin (I-PRF), and CGF within 14 days [56]. The TGF-β1 released by A-PRF has been significantly reduced at 14 days. The TGF-β1 released by CGF was significantly reduced on day 7. The VEGF of A-PRF was also significantly reduced on day 7 (the reduction was greater than 20 pg/mL). The total amount of VEGF released by the two was almost unchanged at 14 days. The total amount of TGF-β1 and VEGF released by I-PGF were both at a low level (< 50 pg/mL) within 14 days. The short release time of growth factors may determine the short-term effect of platelet concentrates. Their long-term effects need to be further explored in clinical studies with large samples and long follow-up time.

Some factors in the studies can contribute to heterogeneity and have a specific impact on the research results: 1, Baseline bone density. Bone density affects osseointegration and implant survival. It has been shown that implants in type IV bone have a lower survival rate than implants in other bone types [57, 58]. The change in ISQ was more significant and lower for implants placed in low-density and high-density bone, respectively [59]. It has a particular impact on the improvement of ISQ after the application of platelet concentrates. With this in mind, we excluded studies on diabetic patients when screening the literature. Nevertheless, the effect of different bone densities in the included studies was not considered. 2, Implant characteristics, including diameter, length, surface, etc. In the study by Kundu et al., the implant with square thread-form showed the ability to promote bone healing while the reverse buttress thread form implant did not [17]. The study by Alhussaini et al. found a correlation between implant diameter and implant stability [6]. 3, Application method of platelet concentrate. Subgroup analysis on implant stability at 1 week after insertion showed that the result of the group dipping implants in platelet concentrates was better than that of the control group (*P* = 0.001). However, the result of only placing them in the implant sites was not statistically significant (*P* = 0.20). The platelet concentrates wrapped on the surface of implants can directly release growth factors at the bone-implant interface and promote the peri-implant bone formation [41]. We suggest that a combination of the two methods might be considered in clinical applications. 4, The preparation method of the same platelet concentrate, such as centrifugal speed and time [6, 60]. In Fujioka-Kobayashi et al.’s study, human fibroblasts' migration and proliferation levels promoted by PRF prepared at low rotational speed were higher than in the study by Alhussaini et al. [6, 61].

## Limitation

This study also has some limitations. Firstly, our study reported the short-term effects of platelet concentrates (implant stability at 1 week and 4 weeks after insertion, MBL at 3 months after insertion). Due to the lack of long follow-up studies, the long-term effects were not explored. Secondly, the proportion of higher-quality studies in the included studies was relatively small. Thirdly, we conducted a mixed analysis of RCTs and CCTs. A subgroup analysis on the study design was also conducted and the results showed that the effects of the two subgroups were both significant. Even though, differences in the results of studies under two different designs need to be considered.

## The direction of future research

Given the above limitations, some suggestions are made for future research. Firstly, randomized controlled trials need to be conducted to compare the effects of three different types in promoting bone repair and osseointegration and find the type with the best effect. Secondly, in clinical application, it is necessary to standardize the preparation and application methods. Third, large-scale, rigorously designed randomized controlled trials are needed to verify further the long-term effects of platelet concentrates.

## Conclusion

Platelet concentrates were introduced to enhance osseointegration, and the effects have been confirmed in many studies. According to the meta-analysis results, platelet concentrates can significantly improve implant stability and reduce marginal bone loss in the short term. Large-scale studies with long follow‐up periods are required to explore their long-term effects. Researchers also need to conduct RCTs to compare the effects of different types in clinical application given their differences in structure and ability to release growth factors.

## Supplementary Information

**Additional file 1**. Search strategy. All details of the search strategy for PubMed, Cochrane Library, EMBASE, and Web of Science.

**Additional file 2**. Reasons for exclusion. Some studies that meet most inclusion criteria and reasons why they were excluded.

## Data Availability

All data generated or analysed during this study are included in this published article and its supplementary information files.

## Abbreviations

PRP: Platelet-Rich Plasma
PRF: Platelet-Rich Fibrin
CGF: Concentrated Growth Factor
PDGF: Platelet-Derived Growth Factor
TGF-β1: Transforming Growth Factor-β1
TGF-β2: Transforming Growth Factor-β2
VEGF: Vascular Endothelial Growth Factor
ISQ: Implant Stability Quotient
PTV: Periotest Value
MBL: Marginal Bone Loss
PRISMA: Preferred Reporting Items for Systematic Reviews and Meta-analyses
PICOS: Participants, Interventions, Comparisons, Outcomes, and Study design
RCTs: Randomized Controlled Trials
CCTs: Clinical Controlled Trials
ROBINS-I: The risk of bias in non-randomized studies—of interventions
MD: Mean Difference
SMD: Standardized Mean Difference
RFA: Resonance Frequency Analysis
CI: Confidence Interval
FGF: Fibroblast Growth Factor
BMP: Bone Morphogenetic Protein
IGF: Insulin-like Growth Factor
PDGF-AB: Platelet-Derived Growth Factor-AB
TGF-1: Transforming Growth Factor-1
IGF-1: Insulin-like Growth Factor-1
PDGF-B: Platelet-Derived Growth Factor-B
A-PRF: Advanced platelet-rich fibrin
I-PRF: Injected platelet-rich fibrin
NR: Not Reported
