# Supplementary material for: Effects of platelet concentrates on implant stability and marginal bone loss: a systematic review and meta-analysis
Source: BMC Oral Health. 2021 Nov 12;21:579. doi: 10.1186/s12903-021-01929-x (PMC8588658; doi:10.1186/s12903-021-01929-x)
Supplement: Supplementary file 1 — Additional file 1. Search strategy. All details of the search strategy for PubMed, Cochrane Library, EMBASE, and Web of Science. [file 12903_2021_1929_MOESM1_ESM.docx]

| Search | Query |
| --- | --- |
| #1 | "Dental Implants"[MeSH Terms] OR "Dental Implantation"[MeSH Terms] OR "dental prosthesis, implant supported"[MeSH Terms] |
| #2 | "implant supported denture*"[Title/Abstract] OR "implant supported dental prostheses"[Title/Abstract] OR "implant supported dental prosthesis"[Title/Abstract] OR "dental prosthesis implantation*"[Title/Abstract] OR "Dental Implantation"[Title/Abstract] OR "dental implant*"[Title/Abstract] OR ("dental"[Title/Abstract] AND "implant*"[Title/Abstract]) |
| #3 | #1 OR #2 |
| #4 | "Platelet-Rich Fibrin"[MeSH Terms] OR "Blood Platelets"[MeSH Terms] OR "Platelet-Rich Plasma"[MeSH Terms] |
| #5 | "platelet*"[Title/Abstract] OR "thrombocyte*"[Title/Abstract] OR "platelet concentrate*"[Title/Abstract] OR "platelet rich plasma*"[Title/Abstract] OR "platelet rich fibrin*"[Title/Abstract] OR "concentrated growth factor*"[Title/Abstract] OR "PRP"[Title/Abstract] OR "PRF"[Title/Abstract] OR "CGF"[Title/Abstract] |
| #6 | #4 OR #5 |
| #7 | #3 AND #6 |

Table 1 Search strategy for Pubmed

| Search | Query |
| --- | --- |
| #1 | "Dental Implants"[MeSH Terms] OR "Dental Implantation"[MeSH Terms] OR "dental prosthesis, implant supported"[MeSH Terms] |
| #2 | "implant supported denture*"[Title/Abstract] OR "implant supported dental prostheses"[Title/Abstract] OR "implant supported dental prosthesis"[Title/Abstract] OR "dental prosthesis implantation*"[Title/Abstract] OR "Dental Implantation"[Title/Abstract] OR "dental implant*"[Title/Abstract] OR ("dental"[Title/Abstract] AND "implant*"[Title/Abstract]) |
| #3 | #1 OR #2 |
| #4 | "Platelet-Rich Fibrin"[MeSH Terms] OR "Blood Platelets"[MeSH Terms] OR "Platelet-Rich Plasma"[MeSH Terms] |
| #5 | "platelet*"[Title/Abstract] OR "thrombocyte*"[Title/Abstract] OR "platelet concentrate*"[Title/Abstract] OR "platelet rich plasma*"[Title/Abstract] OR "platelet rich fibrin*"[Title/Abstract] OR "concentrated growth factor*"[Title/Abstract] OR "PRP"[Title/Abstract] OR "PRF"[Title/Abstract] OR "CGF"[Title/Abstract] |
| #6 | #4 OR #5 |
| #7 | #3 AND #6 |

Table 2 Search strategy for EMBASE

| Search | Query |
| --- | --- |
| #1 | 'tooth implant'/exp OR 'tooth implantation'/exp OR 'implant-supported denture'/exp |
| #2 | 'tooth implant*':ab,ti OR 'implant supported denture*':ab,ti OR 'implant-supported dental prostheses':ab,ti OR 'implant supported dental prosthesis':ab,ti OR 'dental prosthesis implantation*':ab,ti OR 'dental implantation':ab,ti OR 'dental implant*':ab,ti OR ('dental':ab,ti AND 'implant*':ab,ti ) |
| #3 | #1 OR #2 |
| #4 | 'thrombocyte'/exp OR 'thrombocyte rich plasma'/exp OR 'platelet-rich fibrin'/exp |
| #5 | 'platelet*':ab,ti OR 'thrombocyte*':ab,ti OR 'platelet rich plasma*':ab,ti OR 'platelet rich fibrin*':ab,ti OR 'prp':ab,ti OR 'prf':ab,ti OR 'thrombocyte rich plasma*':ab,ti |
| #6 | #4 OR #5 |
| #7 | #3 AND #6 |

| Search | Query |
| --- | --- |
| #1 | "Dental Implants"[MeSH Terms] OR "Dental Implantation"[MeSH Terms] OR "dental prosthesis, implant supported"[MeSH Terms] |
| #2 | ("Implant Supported Dentures"):ti,ab,kw OR ("Implant Supported Dentures"):ti,ab,kw OR ("Implant-Supported Dental Prostheses"):ti,ab,kw OR ("Implant Supported Dental Prosthesis"):ti,ab,kw OR ("Dental Prosthesis Implantation"):ti,ab,kw OR ("Dental Prosthesis Implantations"):ti,ab,kw OR ("Dental Implantation"):ti,ab,kw OR ("Dental Implant"):ti,ab,kw OR ("Dental Implants"):ti,ab,kw OR (("dental"):ti,ab,kw AND (("implant"):ti,ab,kw OR ("implants"):ti,ab,kw)) |
| #3 | #1 OR #2 |
| #4 | "Platelet-Rich Fibrin"[MeSH Terms] OR "Blood Platelets"[MeSH Terms] OR "Platelet-Rich Plasma"[MeSH Terms] |
| #5 | ("platelet"):ti,ab,kw OR ("platelets"):ti,ab,kw OR ("thrombocyte"):ti,ab,kw OR ("thrombocytes"):ti,ab,kw OR ("platelet rich plasma"):ti,ab,kw OR ("platelet rich fibrin"):ti,ab,kw OR ("PRP"):ti,ab,kw OR ("PRF"):ti,ab,kw |
| #6 | #4 OR #5 |
| #7 | #3 AND #6 |

Table 3 Search strategy for Cochrane Library

| Search | Query |
| --- | --- |
| #1 | (((((TS=("implant supported denture")) OR TS=("implant supported dental prostheses")) OR TS=("implant supported dental prosthesis")) OR TS=("dental prosthesis implantation")) OR TS=("Dental Implantation")) OR TS=("dental implant") |
| #2 | (TS=("dental")) OR TS=("implant") |
| #3 | #1 OR #2 |
| #4 | (((((TS=("platelet*")) OR TS=("thrombocyte*")) OR TS=("platelet rich plasma*")) OR TS=("platelet rich fibrin*")) OR TS=("PRP ")) OR TS=("PRF ") |
| #5 | #3 AND #4 |

Table 4 Search strategy for Web of Science
