# Supplementary material for: Effects of platelet concentrates on implant stability and marginal bone loss: a systematic review and meta-analysis
Source: BMC Oral Health. 2021 Nov 12;21:579. doi: 10.1186/s12903-021-01929-x (PMC8588658; doi:10.1186/s12903-021-01929-x)
Supplement: Supplementary file 2 — Additional file 2. Reasons for exclusion. Some studies that meet most inclusion criteria and reasons why they were excluded. [file 12903_2021_1929_MOESM2_ESM.docx]

Table 1 Some excluded studies that meet most inclusion criteria and reasons why they were excluded

| Study | Reason for exclusion |
| --- | --- |
| Ibraheem et al., 2015 [62] | Participants were diabetic patients |
| Malik et al., 2012 [63] | Participants were diabetic patients |
| Ustaoğlu et al., 2020 [64] | The outcomes were implant soft tissue thickness and keratinized tissue width |
| Temmerman et al., 2018 [65] | The outcome was keratinized tissue width |
| Gkikas et al., 2020 [66] | The outcome was buccal bone horizontal dimension |
| Georgakopoulos et al., 2014 [20] | The outcome was temporal texture differentiation associated with the bone formation properties |
| Arakeeb et al., 2019 [67] | The outcome was relative bone density |
| Pal et al., 2018 [68] | The aim was to compare implant stability of two-time use of platelet-rich growth factor with that of one-time use |
